# Automated Identification of Multiple Findings on Brain MRI for Improving Scan Acquisition and Interpretation Workflows: A Systematic Review

**DOI:** 10.3390/diagnostics12081878

**Published:** 2022-08-03

**Authors:** Kaining Sheng, Cecilie Mørck Offersen, Jon Middleton, Jonathan Frederik Carlsen, Thomas Clement Truelsen, Akshay Pai, Jacob Johansen, Michael Bachmann Nielsen

**Affiliations:** 1 Department of Radiology, Copenhagen University Hospital Rigshospitalet, 2100 Copenhagen, Denmark; cecilie.moerck.offersen@regionh.dk (C.M.O.); jonathan.frederik.carlsen@regionh.dk (J.F.C.); ap@cerebriu.com (A.P.); mbn@dadlnet.dk (M.B.N.); 2 Department of Clinical Medicine, University of Copenhagen, 2200 Copenhagen, Denmark; thomas.clement.truelsen@regionh.dk; 3 Department of Computer Science, University of Copenhagen, 2200 Copenhagen, Denmark; jom@cerebriu.com (J.M.); jj@cerebriu.com (J.J.); 4 Cerebriu A/S, 1127 Copenhagen, Denmark; 5 Department of Neurology, Copenhagen University Hospital Rigshospitalet, 2100 Copenhagen, Denmark

**Keywords:** artificial intelligence, machine learning, brain MRI, brain diseases, workflow

## Abstract

We conducted a systematic review of the current status of machine learning (ML) algorithms’ ability to identify multiple brain diseases, and we evaluated their applicability for improving existing scan acquisition and interpretation workflows. PubMed Medline, Ovid Embase, Scopus, Web of Science, and IEEE Xplore literature databases were searched for relevant studies published between January 2017 and February 2022. The quality of the included studies was assessed using the Quality Assessment of Diagnostic Accuracy Studies 2 tool. The applicability of ML algorithms for successful workflow improvement was qualitatively assessed based on the satisfaction of three clinical requirements. A total of 19 studies were included for qualitative synthesis. The included studies performed classification tasks (n = 12) and segmentation tasks (n = 7). For classification algorithms, the area under the receiver operating characteristic curve (AUC) ranged from 0.765 to 0.997, while accuracy, sensitivity, and specificity ranged from 80% to 100%, 72% to 100%, and 65% to 100%, respectively. For segmentation algorithms, the Dice coefficient ranged from 0.300 to 0.912. No studies satisfied all clinical requirements for successful workflow improvements due to key limitations pertaining to the study’s design, study data, reference standards, and performance reporting. Standardized reporting guidelines tailored for ML in radiology, prospective study designs, and multi-site testing could help alleviate this.

## 1. Introduction

Brain magnetic resonance imaging (MRI) is recognized as the imaging modality that produces the best images of brain tissues, body fluids, and fat [1]. It remains the most appropriate modality for diagnosing patients with symptoms of multiple brain diseases including inflammatory diseases, dementia, neurodegenerative disease, cerebrovascular disease, and brain tumors [2,3,4,5]; hence, it plays an important role in multiple clinical scenarios ranging from acute diagnostics to routine follow-ups.

A brain MRI scan typically consists of several scan sequences, the most commonly included being T1-weighted (T1) and T2-weighted (T2) sequences, a diffusion-weighted imaging (DWI) sequence, a fluid attenuated inversion-recovery (FLAIR) sequence, and a bleeding sensitive sequence, e.g., T2* gradient-recall-echo (T2*-GRE) [6]. Selecting the appropriate sequences a priori can be challenging, because many brain diseases often present with the same symptoms [7] while requiring different combinations of sequences for correct diagnosis. Inefficient MR sequence selection can increase the risk of inconclusive scans, scan recalls, and inappropriate usage of gadolinium contrast agents [8]. It can also cause the redundant scanning of patients and result in increased patient inconvenience [8], higher risks of radiologist burnout due to increased workload [9], and prolonged reporting time of potentially time-sensitive diseases [10].

In recent years, machine learning (ML) has been increasingly applied in neuroimaging to alleviate some of these challenges using automated workflow improvements [11]. A potential application of ML algorithms could be to automate scan-sequence acquisition alterations based on real-time image analysis while the patient is still in the scanner [12]. Another application could be to improve scan interpretation efficiencies by prioritizing the reading list of essential and acute/critical findings [11]. However, to improve any existing workflows, ML algorithms require the satisfaction of at least three essential requirements. First, the ML algorithms must be developed and tested in a scenario that reflects clinical practice [13,14]. For improving scan acquisition and interpretation workflows, this means ML algorithms capable of automatically identifying or differentiating between multiple brain diseases with the identification of brain infarcts, hemorrhages, and tumors, being a must due to their frequent and time-critical nature [15,16]. This also requires consecutive datasets not prone to spectrum biases and ground-truth labels unaffected by selection bias [13]. Secondly, tests of the ML algorithm should be performed on an out-of-distribution dataset to account for potential overfitting [13,17]. This could for instance be achieved by testing the algorithms on an external dataset sourced from a different point in time or geographical location compared to the training dataset. Finally, if ML algorithms are to gain widespread trust and usage, their technical performance results should be acceptable with respect to balancing increased workloads from false-positive findings and risk of missing important findings from false-negative results [18]. One method of assessing whether an ML algorithm performs a certain task acceptably could be to compare its performance to that of domain experts performing similar tasks.

Many studies about ML algorithms used within neuroradiology exist [19]. However, few of them address the important question of whether these algorithms could bring actual benefits to clinicians and patients if they were deployed today. To address this knowledge gap, we conducted a systematic review on how well the most recent ML algorithms could identify multiple brain diseases with the aim of evaluating their applicability for improving existing scan acquisition and interpretation workflows based on the satisfaction of the aforementioned requirements.

## 2. Materials and Methods

This systematic review was conducted in accordance with the Preferred Reporting Items for Systematic Reviews and Meta-Analyses (PRISMA) statement [20]. The study protocol was registered in the Prospective Register of Systematic Reviews (PROSPERO) under number CRD42022329801 during the research process. 

### 2.1. Literature Search

The literature was searched in MEDLINE (accessed through PubMed), Ovid Embase, Elsevier Scopus, Clarivate Web of Science, and IEEE Explore in order to find studies covering both clinical and technical aspects of the review question. The search period ranged between 1 January 2017 and 10 February 2022. This relatively short period was selected due to the nature of deep learning research, with rapid development cycles rendering older studies less relevant for the review question. Structured search terms (MeSH, Emtree), such as “magnetic resonance imaging”, “machine learning”, and “brain diseases”, were combined using Boolean operators and supplemented with free keyword search terms, such as “detection”, “classification”, “triaging”, or “workflow”. Multiple additional keywords describing pathologies of interest, such as “neoplasm”, “stroke”, “intraparenchymal hemorrhage”, “subarachnoid hemorrhages”, and “subdural hemorrhages”, were also included in the search string. The full search string can be found in Appendix A.

### 2.2. Study Selection

Records that developed ML algorithms for the automated identification of normal and abnormal brain diseases were screened. Main inclusion and exclusion criteria are listed in Table 1.

Two medical doctors (K.S. and C.M.O.) served as reviewers. They independently screened all records based on title and abstract. This was followed by the extraction of relevant reports for full-text screening and final study inclusion. For the process of record and report screening, Covidence (Melbourne, Australia) was used. Discussions between both reviewers were held to resolve any conflicts, but if a consensus was not reached, a third reviewer (J.F.C.) was consulted.

### 2.3. Data Extraction and Analysis

The reviewers independently extracted data from the included studies according to a pre-defined datasheet. Study and algorithm characteristics were extracted and comprised of the following: (a) study information, (b) population/dataset characteristics including number of patients or images, pathology in the dataset, and MR sequences available, (c) aim of algorithms, (d) type of algorithm, and (e) training and testing strategies including how data-splits were performed. Reported performance metrics together with confidence intervals were also extracted, including accuracy, sensitivity, specificity, F1-score, negative predictive value (NPV), positive predictive value (PPV), and area under the receiver operating characteristic curve (AUC). Furthermore, the Dice score coefficient (DSC), which is one of the most common evaluation metrics used in medical segmentation tasks [21], was extracted where applicable in brain segmentation studies. Performance numbers were summarized using descriptive statistics. If multiple results were reported for different variations of the same algorithm, only the best performance result was extracted unless otherwise stated. When available, performance results were extracted from external test datasets. Included studies were divided by tasks of the included algorithms. The analysis of data was primarily conducted using pivot tables and the in-built analysis tools of Microsoft Excel.

### 2.4. Quality Assessment of Included Studies

The two reviewers independently assessed the quality of the included studies by using the tailored questionnaire Quality Assessment of Diagnostic Accuracy Studies 2 (QUADAS-2) [22] with signaling questions covering risk of bias and concern for applicability in the domains of patient selection, index test, reference test, and flow and timing. For each study, the respective domains were graded as high-, unclear-, or low risk of bias/concern for applicability. Discordance between the reviewers was resolved through discussion.

### 2.5. Evaluation of Applicability for Workflow Improvements

The applicability of each included ML algorithm for improving scan acquisition and interpretation workflows was qualitatively assessed based on three essential requirements previously mentioned in the Introduction (Section 1): (A) reflection of clinical practice, (B) testing on an external out-of-distribution dataset, and (C) acceptable performance results. Each requirement was indicated as being ‘*Satisfied*’ (*S*) or ‘*Not Satisfied*’ (*NS*). The first requirement (A) was satisfied if the patient population was consecutively sampled, if the disease distribution was well reported, and if the study was assessed as having low risk of bias/concern for applicability for the review question. The second requirement (B) was satisfied if external test datasets with data from a different time-period and geographical location were used to produce performance results. Lastly, the third requirement (C) was graded satisfied if a majority of the abovementioned result metrics exceeded a pre-defined threshold of 85% of the maximum attainable value. This threshold for acceptable performance was selected because it reflected the performance levels of a neuroradiologist when performing similar disease identification tasks [23].

## 3. Results

### 3.1. Study Selection and Data Extraction

**Figure 1:**
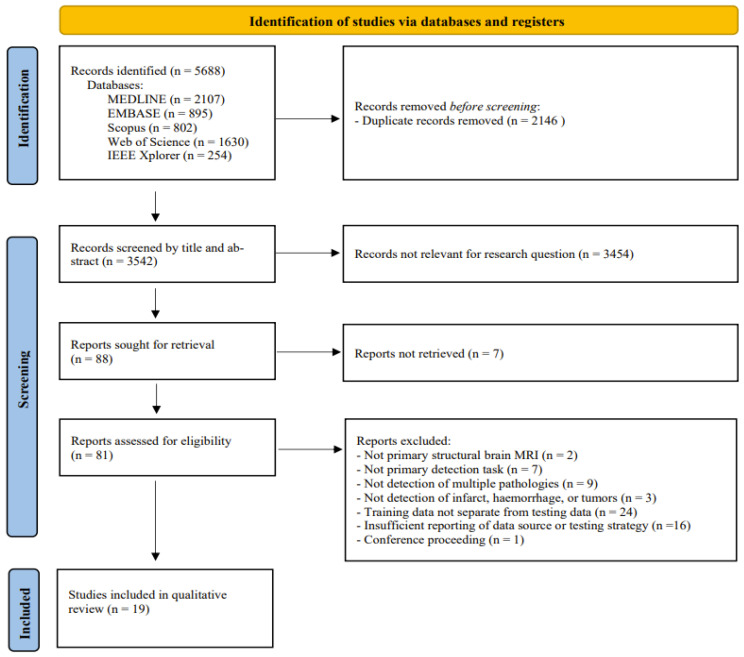
PRISMA workflow: 5688 records screened, and 19 studies were included for qualitative review.

The search of electronic databases returned 5688 records. The removal of duplicates resulted in 3542 records. Screening record titles and abstracts resulted in 81 reports selected for full-text eligibility assessments, of which 19 studies were included for qualitative review. The study inclusion process is illustrated in Figure 1.

Details about the study’s characteristics are summarized in Table 2. All 19 studies included were of retrospective design. Study populations varied with regard to source and size. Twelve out of nineteen studies (63%) used public datasets for development and testing of their algorithms. These datasets included The Whole Brain Atlas from Harvard Medical School (HMS) [24], The Cancer Imaging Archive (TCIA) [25], the Brain Tumor Segmentation (BRATS) challenge dataset [26], and the Ischemic Stroke Lesion Segmentation (ISLES) challenge set [27]. Study populations varied between 100 and 500 patients with one study (5%) having a population of less than 100 patients and five studies (26%) having a population larger than 1000 patients. All large study populations were private, i.e., part of a local in-house dataset not publicly available to researchers outside of the research institution in question. Six studies (31%) only reported on data size as the number of 2D images ranging from 200 images to 4600 images. Training, validation, and testing of algorithms were on average performed using 69%, 3%, and 28% of all available data, respectively. Validation was performed only in six (31%) studies. Testing was mostly performed on data split out from the same data source; however, an external dataset with data from a different time-period and geographical location was used in three (15%) studies.

All studies developed algorithms focusing on brain disease identification using either classification or segmentation tasks. Seven studies (37%) focused on a binary classification of images into either normal/abnormal or differentiation between two diseases, five studies (26%) focused on a multiclass classification of images into specific disease categories, and seven studies (37%) focused on a multiclass segmentation of specific diseases. Most algorithmic tasks employed deep discriminative models, with 14 (74%) studies using convolutional neural networks (CNN). Three (16%) studies employed deep generative models, with two (11%) studies using variational autoencoders (VAE) and one (5%) study using generative adversarial networks (GANs). Reference tests were mostly labels and delineations made by neuroradiologists. Exceptions were found in the study by Wood et al. [46] where reference labels were generated using natural language processing (NLP) of radiological reports and in the study by Ahmadi et al. [28], where reference delineations were constructed using principal component analysis (PCA). Details about the test setup and performance metrics for binary classification, multiclass classification, and segmentation algorithms are summarized in Table 3 and Table 4, respectively.

Different performance measures were reported for each study. For classification studies, the most frequently reported performance metrics were AUC, accuracy, sensitivity, and specificity. AUC ranged from 0.765 to 0.997 while accuracy, sensitivity, and specificity ranged from 80% to 100%, 72% to 100%, and 65% to 100%, respectively. Positive predictive and negative predictive values were reported in nine studies (47%) and ranged from 12% to 94% and 48% to 99%, respectively. The higher performance values were predominantly observed in binary classification studies with a smaller study population, while the lower values were seen when identifying brain tumors. For segmentation studies, the Dice Score Coefficient was the most reported measure ranging from 0.300 for infarct segmentations to 0.912 for glioma and multiple-sclerosis segmentations. Sensitivity and specificity were observed to range from 13% to 99.9% and 87% to 99.8%, respectively, with the lower sensitivity values attributed to brain infarct segmentations.

### 3.2. Applicability to Workflow Improvement

The applicability of the included ML algorithms for improvements in scan acquisition and interpretation workflows was evaluated based on the satisfaction of the three requirements of (A) testing environments reflecting clinical practice, (B) test on external out-of-distribution datasets, and (C) acceptable algorithm performance results; see Section 2. Evaluation results for each requirement are summarized in Table 3 and Table 4 as well. Ten (53%) of nineteen studies were assessed as having acceptable performance; however, only one (5%) satisfied the requirement of being tested in a clinical environment reflecting clinical practice and three (15%) satisfied the requirement of testing on an external out-of-distribution dataset. Three studies (15%) satisfied two main requirements. These studies used privately acquired datasets. No studies satisfied all three main requirements for successful workflow integrations.

### 3.3. Quality Assessment

**Figure 2:**
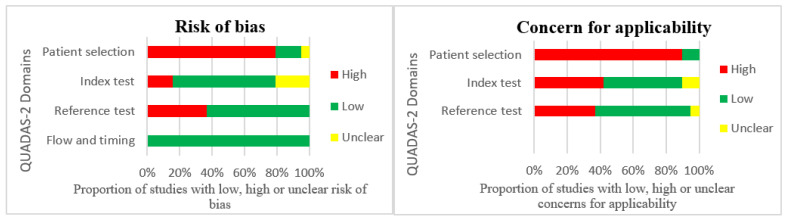
Summary of risk of bias and concern for the applicability of included studies.

The Quality Assessment of Diagnostic Accuracy Studies 2 tool was applied to all included studies in this review. The results of the risk of bias/concern for applicability analysis are presented in Table 5 and summarized in Figure 2.

Significant risks of bias and concern for applicability were seen in the domain of patient selection, index test, and reference. Reasons for these include a lack of consecutive patient populations in eighteen studies, arbitrary classification of equivocal diseases in two studies, large threshold values and the exclusion of smaller lesions in two studies, and automatically generated reference labels in two studies. Only two studies were assessed with low or unclear risk of bias and concern for applicability in all domains.

No meta-analysis was conducted due to inherent heterogeneities in study tasks, population characteristics, and performance metrics.

## 4. Discussion

In this systematic review, we found that the included algorithms varied considerably in terms of tasks, data requirements, and applicability to workflow improvement. With respect to patient selection, index tests, and reference tests, a significant risk of bias was seen. Most (63%) surveyed algorithms were developed using public datasets derived from ML development challenges. This largely explains the following observed patterns: data size restricted to a few hundred patients, specific disease distribution in multiple datasets, and algorithm inference capabilities based on a limited amount of MR scan sequences. T2 and T2-FLAIR were the most frequently used sequences for disease identification of multiple brain diseases. However, this observation might be confounded by the usage of public datasets. Deep neural networks and derivatives thereof were the most frequently applied ML algorithms, which might be due to their proven high performance and robust feature input methods [47]. All studies published in clinical journals used private datasets with larger patient populations. All studies that satisfied more than one workflow applicability requirement likewise used private datasets. This observed pattern of private dataset usage fits into the general trend, where promising ML algorithms are validated and regulatorily approved for clinical usage based on retrospective, unpublished, and often proprietary data from a single institution [48].

Performance results varied considerably as well. About half of the algorithms exceeded the pre-defined threshold of 85% of their respective performance metrics. Disease segmentation performance was generally lower due to the complexity of this task. These results are corroborated by similar reviews performed by Zhang et al. [49] and van Kempen et al. [50] focusing on ischemic stroke and glioma segmentation, respectively. Similar performance levels in relation to triaging performance were also observed across other imaging modalities. Hickman et al. for instance demonstrated pooled AUC, sensitivity, and specificity of 0.89, 75.4%, and 90.6%, respectively, for screening and triaging mammography using machine learning techniques [51], which are in line with what is observed in this review. Hence, consistent performance results are reported across multiple imaging modalities when using similar methods. [19,52]. However, large performance gaps were seen across clinical settings and study designs, partially owing to the well-documented effect of domain shift [53]. For example, Gauriau et al. [33] tested an algorithm with moderately low sensitivity and specificity of 77% and 65%, respectively. These results were, however, attained on a large out-of-distribution dataset with a comprehensive representation of almost all diseases seen in everyday clinical practice. On the other hand, the algorithm developed by Lu, Lu et Zhang [40] achieved a binary classification accuracy, sensitivity, and specificity of 100%, but this was achieved on a very small subset of 87 2D MR-slices split out from the same data source as the training data and not reflecting clinical practice. These findings support the approach of considering multiple requirements for study design, study population, testing strategies, and performance when assessing benefits and limitations of applying ML algorithms into existing workflows.

### 4.1. Potential Benefits of Integrating ML into Existing Scan- and Interpretation Workflows

ML algorithms for clinical workflow integrations have been studied extensively in the past years with multiple authors suggesting different applications [11,12,52,54,55]. Olthof et al. suggest that radiologist workflows could be supported, extended, or replaced by ML functionalities [56].

Based on the findings in this review, scan acquisitions workflows could be supported by multiclass classification and segmentation algorithms. These algorithms, using only a few scan sequences acquired at the beginning of the scan acquisition process, could help classify initial scan images into different disease categories while the patient is still in the scanner and subsequently direct further scan acquisition based on real-time findings. This could prevent the excessive scanning of patients with no significant findings while ensuring fast scan acquisition for stroke patients and appropriate scan acquisition for tumor patients. The fact that 42% of the ML algorithms included in this review could successfully perform multiclass classification and segmentation based on a single MR sequence supports the feasibility of this concept.

Scan interpretation workflows, on the other hand, could be supported by all algorithms in this review. In fact, some of the surveyed binary classification studies aimed explicitly to support interpretation workflows by doing worklist prioritization of critical findings [33,41,45] and, hence, ensure faster reporting times and improved patient outcomes. Multi-class classification and segmentation algorithms could extend this further by offering potential automated diagnosis reporting, biomarker quantification, and even disease progression predictions. None of the surveyed algorithms, however, satisfied all requirements for successful workflow improvements due to key limitations.

### 4.2. Limitations of Included Studies and Future Directions

Important limitations pertaining to study design, data source, model development, and testing methodologies were uncovered using an analysis of the risk of bias/concern for applicability and applicability assessment for workflow improvements. First, patient selections were not consecutive and largely based on public datasets that consisted of imaging cases with high signal-to-noise ratios and selection biases. This is especially true for the BRATS challenge dataset, which is known to have handpicked and well-processed representations of brain gliomas that are very characteristic and visually recognizable, thus resulting in many algorithms achieving good performance when being developed and tested on it [57]. This could potentially introduce an overestimation of model performance and limit integration into clinical practices that face more heterogeneous images of brain diseases. Secondly, index tests were limited by insufficient reporting of model thresholds or deliberately large thresholds chosen for favorable performance reporting. Nael et al. [41], for instance, demonstrated that their model performance dropped significantly when detecting a smaller infarction volume of <0.25 mL compared to volumes of 1 mL. Because the accurate delineation of size, location and development of ischemic lesions have great prognostic implications [58], this trend of size-dependent accuracy could pose challenges to performing accurate recovery predictions and, hence, overall stroke management. Thirdly, reference tests similarly introduced critical biases, especially in the included studies that used 2D-image datasets with handpicked 2D-images and labels as ground truth. This selection of representative images could have introduced priors that are easily exploitable by ML algorithms, as has previously been demonstrated in similar datasets [59]. Fourthly, about half of the surveyed ML algorithms had unacceptably low sensitivity and specificity, which could increase scan acquisition workloads and more worryingly decrease patient safety. Finally, only a minor proportion reported on the clinically relevant metrics of positive and negative predictive values. This, combined with the lack of testing on out-of-distribution datasets, might have presented skewed performance impressions not accounting for all relevant conditions in the intended target population [13].

Future studies developing ML algorithms applicable for workflow improvements should ensure the possession of a consecutive patient population reflecting the desired target population, transparent reporting of patient population characteristics and thresholds for index tests, and performance levels reported through metrics that incorporate different aspects of positive and negative findings. Low false-negative rates should be prioritized, thus ensuring adequate patient safety by having the fewest possible missed findings. Disease prevalence must be considered so as to account for positive and negative predictive values. To alleviate some of these limitations, standardized reporting guidelines tailored for AI in radiology [60], prospective study designs with consecutive patient sampling, and multi-site testing with clinical partners must be considered. The challenges of low sensitivity and specificity might be addressed by rethinking existing data acquisition strategies and model architectures. For instance, temporal information from follow-up scans or contrast-enhancement kinetics can be taken into account. Similar strategies are being used on PET-CT scans resulting in improved tumor classification specificity [61].

### 4.3. Limitations of This Review

This review should be read in view of limitations including publication and reporting bias. We limited our inclusion criteria to studies that could identify multiple brain diseases including brain infarcts, hemorrhages, or tumors, and we further restricted our limitation to studies that have tested their algorithms on data separate from training data. Next, we assessed the applicability of ML algorithms for improving workflows based on a set of requirements not previously validated. All of this might have limited the overview and the impression of this research field. As these criteria were selected based on clinical relevance, the results nonetheless present clinically useful representations of how state-of-the-art ML algorithms could be applied to improve existing scan acquisition and interpretation workflows.

## 5. Conclusions

The surveyed algorithms could potentially support and extend existing workflows. However, limitations pertaining to study design, study data, reference standards, and performance reporting prevent clinical integration. No study satisfied all requirements for successful workflow integration. Standardized reporting guidelines tailored for ML in radiology, prospective study designs, and multi-site testing could help alleviate this. The findings from this review could aid future researchers and healthcare providers by allowing them to critically assess relevant ML studies for workflow improvements and by enabling them to better design studies that validate the benefits of deploying ML in scan acquisition and interpretation workflows.

## Figures and Tables

**Table 1:** Inclusion and exclusion criteria.

Inclusion Criteria:	Exclusion Criteria:
Studies focusing on abnormal brain diseases that included either brain infarct, hemorrhage, or tumor on brain MRI	Studies focusing on tasks not relevant for identification of brain diseases
Studies developing algorithms tested on a dataset that was separate from the training dataset	Studies focusing on identification of a single brain disease only
Peer-reviewed studies in English	Studies focusing on development of ML for specialized MR sequences (e.g., MR elastography, functional MRI) or other imaging modalities (e.g., SPECT, PET, CT, US)
	Studies with primarily non-adult populations
	Editorials, case series, letters, conference proceedings, reviews, and inaccessible papers

**Table 2:** Study population and data characteristics.

Author	Data Source	No. Patients /Images	Training Data	Validation Data	Testing Data	Disease Distribution in Data	MR Sequences Utilized	MR Field Strength
Ahmadi et al., 2021 [28]	Private + Harvard Medical School Whole Brain Atlas	1200 images	1120	N/A	80	12.5% normals 87.5% abnormal incl. glioma, Huntington’s disease, Meningioma, and Alzheimer	2D single slice of: Ax T2	1.5 T
Baur et al., 2021 [29]	Private WMH TCIA	259 patients	100	18	141	42% normal used for unsupervised training 19% multiple sclerosis 15% glioma & glioblastoma 4% microangiopathy 20% WMH	Ax T2-FLAIR	1.5 T 3 T
Duong et al., 2019 [30]	Private	387 patients	295	N/A	92	Normal and 19 different abnormalities incl. MS, high grade glioma, and vascular (acute or subacute ischemia)	Ax T2-FLAIR	1.5 T 3 T
Fayaz et al., 2021 [31]	Harvard Medical School Whole Brain Atlas	4100 images	2870	N/A	1230	50% normal 50% abnormal incl. glioma, meningioma, and Alzheimer	2D single slice of: Ax T2	1.5 T
Felipe Fattori Alves et al., 2020 [32]	Private	67 patients	50	N/A	17	45% inflammatory lesion (incl. MS, vasculitis, toxoplasmosis, pyogenic and septic-embolic brain abscess, etc.) 55% brain tumors (incl. Glioblastoma, anaplastic astrocytoma, anaplastic ependymoma)	Ax T1 & T1 + C Ax T2 Ax T2-FLAIR Ax DWI	1.5 T 3 T
Gauriau et al., 2021 [33]	Private	10,770 patients	7795	473	2502	Normal and 8+ different abnormalities including infarct, hemorrhage, neoplasm, demyelination, and infections	Ax T2-FLAIR	1.5 T 3 T
Gilanie et al., 2018 [34]	Harvard Medical School Whole Brain Atlas	4589 images	3029	N/A	1560	11% normal 89% abnormal incl. cerebrovascular, neoplasm, neurodegenerative, and inflammatory disease	2D single slices of: Ax T1 & T1 + C Ax T2 & Ax PD Ax T2-FLAIR	1.5 T
Han et al., 2020 [35]	OASIS-3 Private	1162 patients	543	N/A	619	47% normals used for unsupervised training 19% normals used for testing 21% dementia of varying degree 7% brain metastasis 6% various disease incl. small infarct, hemorrhage, and white matter lesions	Ax T1 & Ax T1 + c	1.5 T 3 T
Hu et al., 2020 [36]	BRATS 2019 ISLES 2017	459 patients	317	N/A	142	84% glioma (HGG, LGG) 16% acute & subacute infarct	Ax T1 & T1 + C Ax T2 Ax T2-FLAIR Ax DWI, ADC, perfusion	1.5 T 3 T
Kamnitsas et al., 2017 [37]	Private BRATS 2015 ISLES 2015	509 patients	348	N/A	161	75% tumor (high grade glioma, low grade glioma) 13% acute & subacute infarct 12% traumatic brain injury	Ax or Sag T1 & T1 + C Ax T2 & Ax PD Ax T2-FLAIR Ax T2 * GRE Ax DWI & ADC	1.5 T 3 T
Kim et al., 2021 [38]	BRATS 2019 ISLES 2015	259 patients	239	N/A	26	36% normal 60% glioma 4% acute & subacute infarct	2D slices of Ax T1 & T1 + C Ax T2 Ax T2-FLAIR Ax DWI, ADC, perfusion	1.5 T 3 T
Lu et al., 2021 [39]	Private	7134 patients	* 5002	1061	1071	13% acute/subacute stroke 87% non-stroke abnormalities incl. tumor, hemorrhage and normals	Axial T2-FLAIR Axial DWI + ADC	1.5 T 3 T
Lu, Lu et Zhang., 2019 [40]	Harvard Medical School Whole Brain Atlas	291 images	204	N/A	87	39% normal 61% abnormal incl. neoplasm, neurodegenerative, and inflammatory disease	2D single slice of: Ax T2	1.5 T
Nael et al., 2021 [41]	Private	13,215 patients	9845	1248	2122	17% normal 11% acute infarction 5% acute hemorrhage 4% intracranial mass effect 63% other abnormalities including white matter lesions	Ax or Sag T1 & T1 + C Ax T2 Ax T2-FLAIR Ax ADC & DWI Ax T2 * GRE	1.5 T 3 T
Nayak et al., 2020 [42]	Harvard Medical School Whole Brain Atlas &	275 images	165	N/A	110	20% normal 20% stroke 20% neurodegenerative 20% infectious 20% neoplasm	2D single slice of: Ax T2	1.5 T
Nayak et al., 2020 [43]	Havard Medical School	200 images	120	N/A	80	20% normal 20% stroke 20% neurodegenerative 20% infectious 20% neoplasm	2D single slice of: Ax T2	1.5 T
Pereira et al., 2019 [44]	BRATS 2013 BRATS 2017 ISLES 2017	471 patients	358	10	103	89% tumor (high grade glioma, low grade glioma) 11% acute & subacute infarct	Ax T1 & T1 + C Ax T2 Ax T2-FLAIR Ax DWI, ADC, perfusion	1.5 T 3 T
Rauschecker et al., 2020 [23]	Private	178 patients	86	N/A	92	19 different abnormalities incl. MS, high grade glioma, and vascular (acute or subacute ischemia)	Ax T1 + C Ax T2 Ax T2-FLAIR Ax T2 * GRE Ax DWI & ADC	1.5 T 3 T
Wood et al., 2022 [45]	Private	71,206 patients	53,409	9425	7372	Normal and 90+ different abnormalities including vascular disease, neoplasms, demyelination, and atrophy	Ax T2-FLAIR Ax DWI	1.5 T 3 T

Abbreviations: WMH = White Matter Hyperintensity Challenge; BRATS = Brain Tumor Segmentation Challenge Data; HGG = High Grade Glioma; LGG = Low Grade Glioma; ISLES = Ischemic Stroke Lesion Segmentation; OASIS-3 = Open Access Series of Imaging Studies; Ax. = axial. Private = local in-house dataset not publicly available. * Train/test distribution only reported for data pertaining subpart of study performing case-level classification of stroke/not stroke. Disease frequencies in the study populations were also varied with diverse samples of cerebrovascular disease, brain tumors, inflammatory disease, neurodegenerative diseases, neuroinfectious diseases, dementia, traumatic brain injury, and various less significant pathologies. All study populations had samples of brain tumors, while 11 (58%) studies had samples of both normal scans, brain infarcts, hemorrhages, and tumors. The brain pathologies were identified on MR sequences found in typical routine acquisitions with T1, T2, and T2-FLAIR being the most prevalent. Eight studies (42%) used only a single scan sequence, either T2 or T2-FLAIR, for disease inference.

**Table 3:** (**a**) Performance results of binary classification algorithms. (**b**) Performance results of multiclass classification algorithms.

Author	Aim of Algorithm	Type of Algorithm	Ground Truth	Testing Strategy	Performance Results							Workflow Applicability
					AUC	Acc (%)	Sens (%)	Spec (%)	F1 (%)	PPV (%)	NPV (%)	
(**a**)												
Fayaz et al., 2021 [31]	Binary classification of normal and abnormal	CNN + DWT	Expert labels	Train-test split	0.997	N/A	99.7	N/A	N/A	N/A	N/A	(A) Reflecting clinical practice: NS (B) External validation: NS (C) Performance: S *Note: High performance observed on single 2D MR slices*
Felipe Fattori Alves et al., 2020 [32]	Binary classification of inflammatory lesions and brain tumors	RF SVM k-NN	Expert delineation	Train-test split	* 0.906	* 82.7	* 91.2	N/A	* 87.5	N/A	N/A	(A) Reflecting clinical practice: NS (B) External validation: NS (C) Performance: S
Gauriau et al., 2021 [33]	Binary classification of normal and abnormal	CNN	Radiological report	Train-test split incl. external test set	0.800 [0.770; 0.820]	N/A	77.0 [75; 80]	65.0 [61; 69]	78.0 [76; 80]	N/A	N/A	(A) Reflecting clinical practice: S (B) External validation: S (C) Performance: NS
Gilanie et al., 2018 [34]	Binary classification of normal and abnormal	Gabor filter SVM	Expert labels	Train-test split	0.970	96.5	98.0	92.0	N/A	N/A	N/A	(A) Reflecting clinical practice: NS (B) External validation: NS (C) Performance: S
Lu et al., 2021 [39]	Binary classification of stroke/non- stroke patients	CNN + Gating attention mechanism ranking of multi-contrast MRI	Expert labels	Train-test split	** 0.881	N/A	N/A	N/A	N/A	N/A	N/A	(A) Reflecting clinical practice: NS (B) External validation: NS (C) Performance: S
Lu, Lu et Zhang., 2019 [40]	Binary classification of normal and abnormal	CNN + transfer learning	Expert labels	Train-test split	N/A	100.0	100.0	100.0	N/A	N/A	N/A	(A) Reflecting clinical practice: NS (B) External validation: NS (C) Performance: S *Note: High test performance result on small test set <100 2D MR slices*
Wood et al., 2022 [45]	Binary classification of normal and abnormal	Ensemble CNN	NLP labelled radiological report	Train-test split incl. external test set	0.948 [0.945; 0.951]	N/A	91.9 [89.9; 93.9]	84.2 [82.2; 86.2]	92.3 [90.3; 94.3]	N/A	N/A	(A) Reflecting clinical practice: NS (B) External validation: S (C) Performance: S
(**b**)												
Han et al., 2020 [35]	Multiple binary classification of normal/clinical dementia (Dem), normal/brain metastasis (BM), and normal/various diseases (VD) incl. small infarct and hemorrhage.	Unsupervised GAN + 7 Self-attention (SA) modules	Expert label	Train-test split	Dem: 0.765 BM: 0.921 VD: 0.613	N/A	N/A	N/A	N/A	N/A	N/A	(A) Reflecting clinical practice: NS (B) External validation: NS (C) Performance: NS
Nael et al., 2021 [41]	Multiple binary classification of normal/any abnormalities (abn), infarct (inf)/non-infarct, hemorrhage (hem)/non-hemorrhage, and mass effect (ME)/non-mass effect	CNN	Radiological report Expert image delineation	Train-test split incl. external test set	Abn: 0.880 Inf.: 0.970 Hem.: 0.830 ME: 0.870	Abn: 80.0 Inf.: 95.0 Hem: 87.0 ME: 81.0	Abn: 80.0 Inf.: 90.0 Hem.: 72.0 ME: 79.0	Abn: 80.0 Inf.: 97.0 Hem.: 88.0 ME: 81.0	N/A	Abn: 94.0 Inf.: 92.0 Hem.: 32.0 ME: 12.0	Abn: 48.0 Inf.: 96.0 Hem.: 98.0 ME: 99.0	(A) Reflecting clinical practice: NS (B) External validation: S (C) Performance: S
Nayak et al., 2020 [42]	Multiclass classification of normal, stroke, tumor, infectious, degenerative	CNN	Expert labels	Train-test split	N/A	*** 97.5	N/A	N/A	N/A	N/A	N/A	(A) Reflecting clinical practice: NS (B) External validation: NS (C) Performance: S *Note: High test performance result on small test set of 2D MR slices*
Nayak et al., 2020 [43]	Multiclass classification of normal, stroke, tumor, infectious, degenerative	CNN + ELM	Expert labels	Train-test split	N/A	93.8	N/A	N/A	N/A	N/A	N/A	(A) Reflecting clinical practice: NS (B) External validation: NS (C) Performance: S *Note: High test performance result on small test set <100 2D MR slices*
Rauschecker et al., 2020 [23]	Multiclass classification of 19 brain diseases incl multiple sclerosis (MS), high grade glioma, and vascular infarct defined as correctly classified within top 3 differential diagnosis	CNN + Bayesian inference	Expert labels	Train-test split	0.920 [0.880; 0.950]	91.0 [84; 96]	N/A	N/A	N/A	N/A	N/A	(A) Reflecting clinical practice: NS (B) External validation: NS (C) Performance: S

(**a**) Abbreviations: AUC = area under receiver operatic characteristics curve; Acc = accuracy; Sens = sensitivity; Spec = specificity; PPV = positive predictive value; NPV = negative predictive value; S = satisfied; NS = Not Satisfied (see Section 2); CNN = convolutional neural network; VAE = variational auto-encoder; ELM = extreme learning machine; GAN = generative adversarial network; RF = Random forest; SVM = Support vector machine; k-NN = k-Nearest Neighbor; DWT = Deep Wavelet Transform. * Metrics are reported for a random forest classifier on only T1 images ** Results reported for subpart of study performing case-level classification of stroke/not stroke *** Metrics reported for the largest data subset available in the study (**b**) Abbreviations: ELM = extreme learning machine; GAN = generative adversarial network.

**Table 4:** Performance results of segmentation algorithms.

Author	Aim of Algorithm	Type of Algorithm	Ground Truth	Testing Strategy	Performance Results					Workflow Applicability
					DSC	Sens (%)	Spec (%)	PPV (%)	NPV (%)	
Ahmadi et al., 2021 [28]	Multiclass segmentation incl. neoplasm and neurodegenerative disease	CNN	Synthetic labels via robust PCA	Train-test split	0.912	99.9	99.8	N/A	N/A	(A) Reflecting clinical practice: NS (B) External validation: NS (C) Performance: S
Baur et al., 2021 [29]	Multiclass segmentation of normal, MS, glioblastoma (GBM), glioma, microangiopathy (MA), and WMH	Unsupervised VAE	Radiological report Expert image delineation	Train-test split	MS: 0.650 GBM: 0.390 Glioma: 0.350 MA: 0.730 WMH: 0.450	MS: 62.0 GBM: 56.0 Glioma: 29.0 MA: 36.0 WMH: 13.0	N/A	MS: 67.0 GBM: 14.0 Glioma: 28.0 MA: 17.0 WMH: 49.0	N/A	(A) Reflecting clinical practice: NS (B) External validation: NS (C) Performance: NS
Duong et al., 2019 [30]	Multiclass segmentation of 19+ different abnormalities incl. MS, high grade glioma, and infarcts	CNN	Expert image delineation	Train-test split	0.789 [0.767; 0.811]	76.7 [74.2; 79.2]	99.9 [99; 99]	76.9 [75.1; 78.7]	99.0 [99; 99]	(A) Reflecting clinical practice: NS (B) External validation: NS (C) Performance: NS
Hu et al., 2020 [36]	Multiclass segmentation of infarct and glioma	CNN	Expert image delineation	Train-test split	Infarct: 0.300 [0.120; 0.520] Glioma: 0.860	Infarct: 43.0 [16; 70] Glioma: 87.0	Infarct: N/A Glioma: 87.0	Infarct: 35.0 [8; 62] Glioma: N/A	N/A	(A) Reflecting clinical practice: NS (B) External validation: NS (C) Performance: NS
Kamnitsas et al., 2017 [37]	Multiclass segmentation of infarct, traumatic brain injury (TBI), and glioma	Ensemble CNN	Expert image delineation	Train-test split	Infarct: 0.590 [0.280; 0.900] TBI: 0.645 [0.480; 0.810] Glioma: 0.849	Infarct: 60.0 [33; 87] TBI: 63.9 [47; 81] Glioma: 87.7	N/A	Infarct: 68.0 [35; 100] TBI: 69.8 [52; 88] Glioma: 85.3	N/A	(A) Reflecting clinical practice: NS (B) External validation: NS (C) Performance: NS
Kim et al., 2021 [38]	Multiclass segmentation of infarct and glioma	Unsupervised VAE	Expert image delineation	Train-test split	Infarct: 0.278 [0.273; 0.283] Glioma: 0.692 [0.686; 0.698]	Infarct: 42.9 [42.2; 43.6] Glioma: 67.5 [65.1; 69.9]	N/A	Infarct: 20.5 [19.8; 21.2] Glioma: 71.1 [67.2; 75.0]	N/A	(A) Reflecting clinical practice: NS (B) External validation: NS (C) Performance: NS
Pereira et al., 2019 [44]	Multiclass segmentation of infarct incl. penumbra and glioma	CNN	Expert image delineation	Train-test split	Infarct: 0.340 [0.140; 0.540] Penumbra: 0.820 [0.730; 0.910] Glioma: 0.866	Infarct: 55.0 [25; 85] Glioma: 84.6	N/A	Infarct: 36.0 [11; 61] Glioma: 89.8	N/A	(A) Reflecting clinical practice: NS (B) External validation: NS (C) Performance: NS

Abbreviations: VAE = variational auto-encoder; CNN = convolutional neural network; DSC = Dice-score coefficient; Sens = sensitivity; Spec = specificity; PPV = positive predictive value; NPV = negative predictive value; S = satisfied; NS = Not Satisfied; CNN = convolutional neural network; VAE = variational autoencoder; PCA = principal component analysis; MS = multiple sclerosis; WMH = white matter hyperintensities.

**Table 5:** Presentation of risk of bias/concern for applicability analysis results.

Source	Risk of Bias				Concern for Applicability		
	Patient Selection	Index Test	Reference Test	Flow and Timing	Patient Selection	Index Test	Reference Test
Ahmadi et al., 2021 [28]	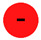	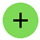	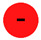	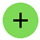	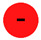	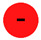	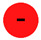
Baur et al., 2021 [29]	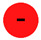	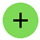	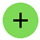	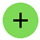	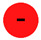	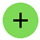	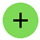
Duong et al., 2019 [30]	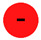	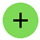	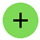	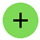	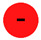	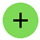	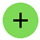
Fayaz et al., 2021 [31]	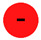	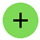	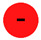	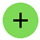	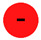	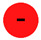	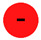
Felipe Fattori Alves et al., 2020 [32]	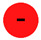	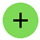	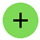	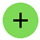	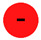	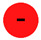	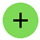
Gauriau et al., 2021 [33]	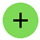	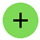	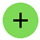	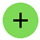	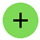	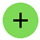	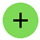
Gilanie et al., 2018 [34]	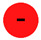	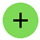	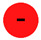	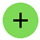	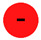	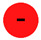	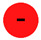
Han et al., 2020 [35]	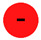	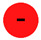	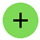	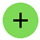	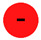	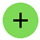	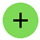
Hu et al., 2020 [36]	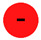	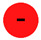	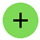	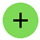	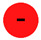	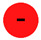	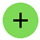
Kamnitsas et al., 2017 [37]	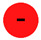	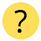	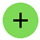	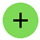	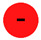	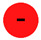	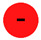
Kim et al., 2021 [38]	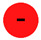	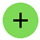	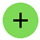	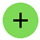	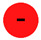	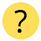	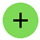
Lu et al., 2021 [39]	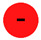	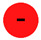	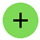	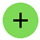	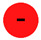	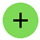	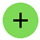
Lu, Lu et Zhang., 2019 [40]	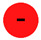	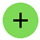	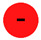	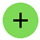	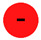	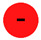	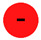
Nael et al., 2021 [41]	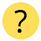	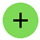	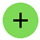	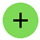	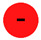	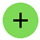	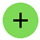
Nayak et al., 2020 [42]	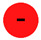	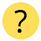	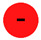	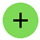	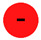	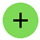	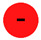
Nayak et al., 2020 [43]	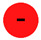	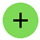	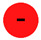	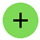	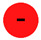	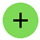	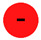
Pereira et al., 2019 [44]	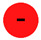	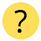	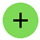	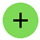	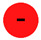	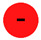	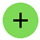
Rauschecker et al., 2020 [23]	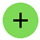	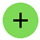	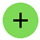	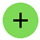	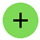	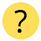	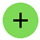
Wood et al., 2022 [45]	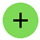	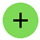	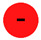	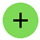	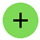	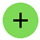	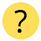

Summary of risk of bias and concern for applicability for all included studies. 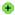 = low risk of bias and concern for applicability; 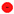 = high risk of bias and concern for applicability; 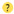 = unclear risk of bias and concern for applicability.

## Data Availability

Data are available upon request.

## Appendix A

**Search strings for databases**

**MEDLINE (PubMed)**

((“magnetic resonance imaging” [MeSH Terms] OR “Multiparametric Magnetic Resonance Imaging” [MeSH Terms] OR “Diffusion Magnetic Resonance Imaging” [MeSH Terms] OR “MRI” [All Fields] OR “MR imag*” [All Fields])

AND

(“Artificial Intelligence” [MeSH Terms] OR “Machine Learning” [MeSH Terms] OR “Deep Learning” [MeSH Terms] OR “Supervised Machine Learning” [MeSH Terms] OR “Artificial Intelligence” [All Fields] OR “Deep Learning” [All Fields] OR “Neural Network” [All Fields] OR “Convolutional Neural Network” [All Fields])

AND

(“Central Nervous System Diseases” [MeSH Terms] OR “Brain Diseases” [MeSH Terms] OR (“brain” [All Fields] AND “neoplasm*” [All Fields]) OR (“brain” [All Fields] AND “tumor*” [All Fields]) OR (“brain” [All Fields] AND “hemorrhage” [All Fields]) OR (“intraparenchymal” [All Fields] AND “hemorrhage” [All Fields]) OR (“subdural” [All Fields] AND “hemorrhage” [All Fields]) OR (“subarachnoid” [All Fields] AND “hemorrhage” [All Fields]) OR (“epidural” [All Fields] AND “hemorrhage” [All Fields]) (“brain” [All Fields] AND “infarct”[All Fields]) OR “brain” [All Fields])

AND

(“anomal*” [All Fields] OR “abnormal*” [All Fields] OR “patholog*” [All Fields] OR “multi-class” [All Fields] OR “critical findings” [All Fields] OR “triag*” [All Fields] OR “automat*” [All Fields] OR “classification” [MeSH Terms] OR “detect*” [All Fields]))

**EMBASE**

exp magnetic resonance imaging/

exp brain disease/

exp machine learning/

exp classification/or detection.mp.

(abnormal or patholog$ or multi-class or critical finding$ or triag$ or automat$).mp. [mp = title, abstract, heading word, drug trade name, original title, device manufacturer, drug manufacturer, device trade name, keyword heading word, floating subheading word, candidate term word]

1 and 2 and 3 and 4 and 5

**Scopus**

(TITLE-ABS-KEY (“*magnetic resonance imaging*” OR “*Multiparametric Magnetic Resonance Imaging*” OR “*MRI*”)) AND (TITLE-ABS-KEY (“*Artificial Intelligence*” OR “*Machine Learning*” OR “*Deep Learning*” OR “*Neural Network*” OR “*Convolutional neural network*”)) AND (TITLE-ABS-KEY (*brain* AND *disease* OR *brain* AND *infarct* OR *brain* AND *hemorrhage* OR *brain* AND *neoplasm** OR *brain* AND *tumor* OR *brain* AND *anomal** OR *brain* AND *abnormal** OR *brain* AND *patholog** OR *brain* “*multi-class*” OR *brain* “*critical finding**” OR *brain* AND *triag** OR *brain* AND *automat**)) AND (TITLE-ABS-KEY (*classification* OR *detection*)) AND (LIMIT-TO (PUBYEAR, *2022*) OR LIMIT-TO (PUBYEAR, *2021*) OR LIMIT-TO (PUBYEAR, *2020*) OR LIMIT-TO (PUBYEAR, *2019*) OR LIMIT-TO (PUBYEAR, *2018*) OR LIMIT-TO (PUBYEAR, *2017*)) AND (LIMIT-TO (DOCTYPE, “*ar*”) OR LIMIT-TO (DOCTYPE, “*re*”))

**Web of Science**

ALL = ((“magnetic resonance imaging” OR “Multiparametric Magnetic Resonance Imaging” OR “MRI”) AND (“Artificial Intelligence” OR “Machine Learning” OR “Deep Learning” OR “Neural Network” OR “Convolutional neural network”) AND (brain disease OR brain anomal* OR brain abnormal* OR brain patholog* OR brain “multi-class” OR brain “critical finding*” or brain triag* OR brain automat* OR brain infarct OR brain hemorrhage OR “intraparenchymal hemorrhage” OR brain neoplasm OR brain tumor) AND (classification OR detection))

**IEEE Xplore**

(“magnetic resonance imaging” OR “Multiparametric Magnetic Resonance Imaging” OR “MRI”) AND (“Artificial Intelligence” OR “Machine Learning” OR “Deep Learning” OR “Neural Network” OR “Convolutional neural network”) AND (brain disease OR brain anomaly OR brain abnormality OR brain pathology OR brain “multi-class” OR brain “critical finding*” OR brain triage OR brain infarct OR brain hemorrhage OR brain neoplasm OR brain tumor) AND (classification OR detection)
